# The Influence of Tear Film Quality on Visual Function

**DOI:** 10.3390/vision8010008

**Published:** 2024-02-26

**Authors:** Snježana Kaštelan, Ksenija Gabrić, Maša Mikuličić, Danijela Mrazovac Zimak, Mirela Karabatić, Antonela Gverović Antunica

**Affiliations:** 1 Department of Ophthalmology, Fundamentals of Medical Skills, School of Medicine, University of Zagreb, Clinical Hospital Dubrava, 10 000 Zagreb, Croatia; 2 Optomed d.o.o., 10 000 Zagreb, Croatia; ksenija.gabric@gmail.com (K.G.); masa.mikulicic@gmail.com (M.M.); 3 Department of Ophthalmology, University Hospital Centre Zagreb, 10 000 Zagreb, Croatia; danijela.mrazovac@gmail.com; 4 Department of Optometry, University of Applied Sciences Velika Gorica, 10 410 Velika Gorica, Croatia; mirela.karabatic@vvg.hr; 5 Department of Ophthalmology, Clinical Nursing, University of Dubrovnik, General Hospital Dubrovnik, 20 000 Dubrovnik, Croatia; agantonela@net.hr

**Keywords:** tear film dysfunction, dry eye disease, visual function, post-blink blur time, tear break-up time, screening test

## Abstract

Background: The prevalence of dry eye disease (DED) is increasing globally, resulting in a variety of eye symptoms characterized by discomfort and visual disturbances. The accurate diagnosis of the disease is often challenging and complex, requiring specialized diagnostic tools. This study aimed to investigate the impact of tear film instability on visual function and to evaluate the value of post-blink blur time (PBBT) as an alternative method for assessing tear film stability. Methods: The study included 62 subjects: 31 with subjective symptoms of DED (Group A) and a control group consisting of 31 healthy participants (Group B). Symptoms were assessed using the standard Schein questionnaire, supplemented with additional questions. PBBT was measured using standard Snellen charts to investigate a potential association between PBBT and tear film dysfunction. Additional clinical assessments included tear film break-up time (TBUT). Results: Statistically significant differences were observed in the average values of PBBT and TBUT between the examined groups. The average PBBT was 8.95 ± 5.38 s in the group with DED and 14.66 ± 10.50 s in the control group, *p* < 0.001. Group A exhibited an average TBUT of 4.77 ± 2.37 s, while Group B had a TBUT of 7.63 ± 3.25 s, *p* < 0.001. Additionally, a strong positive correlation was identified between PBBT and TBUT values (r = 0.455; *p* < 0.001). Conclusions: The research confirms that tear film stability has an important role in the refraction of light and the maintenance of optical quality of vision. PBBT could potentially function as an objective and clinically significant screening test for DED.

## 1. Introduction

Dry eye disease (DED) is a prevalent multifactorial eye condition caused by insufficient tear production or excessive tear evaporation [1,2,3]. It can lead to damage of the eye surface with symptoms such as irritation, redness, itchiness, dryness, fatigue, and foreign body sensation [3,4,5,6,7]. DED is a significant public health concern and one of the leading causes of seeking ophthalmological care [5,7]. Ocular discomfort and pain are the most prevalent complaints in patients with DED and represent a central feature of the condition that significantly affects the quality of life (QoL) [7]. Additionally, individuals with DED may also experience impaired visual acuity and blurred vision [8]. Complaints are more common among contact lens wearers than in the general population [9].

The prevalence of DED varies widely across studies, ranging from 5% to 50% [3,7,10,11,12,13,14,15]. Risk factors for DED include female gender, older age, pregnancy, postmenopausal estrogen therapy, thyroid disease, digital device usage, and contact lens wear. Other factors include refractive surgery, vitamin A deficiency, certain medications, and autoimmune and systemic diseases [3,4,7,16,17,18,19,20,21,22,23,24,25,26]. The significance of DED is expected to rise in the future due to factors such as an aging population, environmental influences, and increased usage of digital devices [10,15].

The diagnosis of DED involves risk factor assessment, self-reported symptoms, patient-reported outcome (PRO) questionnaires, and clinical examinations. Clinical examinations for DED diagnosis include Meibomian gland evaluation, tear break-up time test (TBUT) as an indicator of tear film stability, Schirmer test to measure tear production, corneal and conjunctival staining to assess ocular surface damage, and tear osmolarity measurement [3,7,27,28,29,30,31]. For diagnosing and evaluating the severity of DED symptoms, various PRO questionnaires, including Schein’s questionnaire, are available [16,31,32,33]. However, there is often a discrepancy between the severity of reported dry eye symptoms and the results of clinical tests, making accurate diagnosis challenging [28,32,33]. Recently, physician-scientists specializing in DED collectively established a clinical consensus regarding its definition. One of the significant clinical implications is the recognition that tear film stability serves as a sensitive measure of tear dysfunction. It is an easily measurable, clinically practical, and reproducible marker of tear dysfunction and DED. This underscores the importance of incorporating tear film stability as a key criterion in the clinical definition and diagnostics of DED [7]. 

The tear film has multiple functions, maintaining an optically uniform surface, lubricating eye tissues, and protecting against infections. Efficient tear film production, secretion, and elimination are vital for ocular surface health. Blinking, a physiological function, ensures tear film continuity, distributing it over the cornea and conjunctiva to preserve eye moisture and protect the eye against irritants [10,34]. The altered blinking pattern is prevalent in DED and contributes to its pathology [35]. Extended periods without blinking cause tear film breakage, leading to discomfort and reflexive blinking. In individuals with dry eyes, the less stable tear film results in a rapid break-up, impacting visual function and accelerating symptom onset. Tears, in conjunction with the anterior surface of the cornea, provide approximately 80% of the refractive power of the eye [35,36]. Maintaining a physiologically complete tear film is crucial for normal vision, as any deterioration affects contrast sensitivity and increases optical aberrations, compromising retinal image quality in eyes with tear film instability [37,38,39,40]. Dysfunction can alter best-corrected visual acuity (BCVA), and more importantly for everyday life, functional visual acuity (FVA), highlighting the tear film’s vital role in preserving high-quality vision and QoL [40,41,42]. 

Currently, the instability of the tear film is recognized as an important mechanism in DED [10]. Therefore, measuring the post-blink blur time (PBBT), which indicates the time in seconds for a change in BCVA following a blink, may serve as a simple method to assess DED. This study aims to evaluate the impact of tear film instability on visual function and determine the effectiveness of PBBT as an alternative option for assessing tear film stability, a key clinical indicator of dry eye.

## 2. Materials and Methods

### 2.1. Methods

This cross-sectional, single-center study was approved by the independent local Ethics Committee of the Specialty Eye Hospital Svjetlost and adhered to the tenets of the Declaration of Helsinki. The study participants received both written and verbal information about the study and provided written informed consent before enrollment.

### 2.2. Patients

The study included 62 Caucasian subjects (124 eyes) aged 18 to 79, who underwent routine eye exams at the Specialty Eye Hospital Svjetlost. 

The participants, aged 18 and above, had no pre-existing eye conditions or trauma that could directly impact the conducted test outcomes. All participants exhibited distance BCVA of 20/20 (1.0 in decimal), Schirmer test I (without anesthesia) values within normal limits, and negative corneal and conjunctival staining in both eyes. Subjects with both myopia and hypermetropia up to 1 dioptre sphere were included while those with astigmatism were excluded from the study. Visual acuity and PBBT for this study’s purposes were exclusively examined at a distance. 

Subjects with a history of eyelid disorders, ocular trauma, ocular surgery, ocular infections, corneal scarring, Meibomian gland dysfunction, abnormalities of the nasolacrimal drainage apparatus, and those with permanent or temporary occlusion of lacrimal puncta, as well as individuals who wore contact lenses, were excluded from the study. Additional non-inclusion criteria encompassed glaucoma, cataracts, uveitis, retinal diseases, systemic dry eye-related diseases such as Sjögren’s syndrome, Stevens–Johnson syndrome, rheumatism, cognitive and mental disorders, as well as the use of tear-influencing medications such as antihistamines or psychiatric medications. 

### 2.3. Clinical Evaluations

In addition to collecting the relevant data from the clinical history and completing the modified Schein questionnaire, all participants underwent a standard ophthalmological examination. The examinations were performed by a single investigator in the same examination room under identical conditions to ensure consistency. The room temperature was kept at 22–23 °C and humidity at 40–50% with standard lighting. The investigator had no prior knowledge of Schein’s questionnaire results. Before subjective visual acuity testing, all patients underwent refraction using Nidek ARK-1s auto refracto/keratometer (Nidek Co., Ltd., Gamagori, Japan), Subjective visual acuity testing using digital acuity system Clear Chart 4 (Reichert®, Buffalo, NY, USA) with Snellen optotypes at a standard distance of 6 m and slit lamp examination (Haag-Streit BP 900 Unit, Haag-Streit AG, Köniz, Switzerland). To minimize the potential influence of previous tests, clinical evaluations were conducted in a specific order with a 10-minute interval between each test. The diagnostic tests included photopic distance BCVA, PBBT, TBUT, and slit-lamp examination. The examiner recorded the time of TBUT and PBBT using a digital stopwatch. The performance of clinical tests is described in more detail in the following text.

Participants were divided based on the outcomes of the modified Schein questionnaire. Individuals exhibiting any subjective dry eye symptoms and obtaining a total score of 1 or more were assigned to the Symptomatic group (Group A) whereas those without any symptoms and having a total score of 0 were categorized into the Asymptomatic group (Group B).

### 2.4. Modified Schein Questionnaire

Patients’ subjective symptoms were evaluated using the Schein questionnaire which includes the following six questions [16,33]:

- Do your eyes ever feel dry?
- Do you ever feel a gritty or sandy sensation in your eye?
- Do your eyes ever have a burning sensation?
- Are your eyes ever red?
- Do you notice much crusting on your lashes?
- Do your eyes ever get stuck shut in the morning?

Additional questions in our research included:

- Do you experience disturbances while working on the computer?
- Do you observe changes in your visual acuity after spending extended periods at the computer, watching TV, driving a car, or staying in an air-conditioned room?

These questions were used in the present study to grade subjective symptoms of participants using a slight modification of the scale: numeric values to the responses were added as well as the new category “never”, and ordinal grades were assigned to the possible answers (“never” = 0 points, “rarely” = 1 point, “sometimes” = 2 points, “often” = 3 points, and “all of the time” = 4 points). The questionnaire provided a total numerical score, with a minimum of 0 points for individuals with no symptoms and a maximum of 24 points for those with severe symptoms. 

### 2.5. Post Blink Blur Time 

PBBT represents the time it takes for the change in BCVA following blinking. After measuring distance BCVA using standard Snellen charts, the participant was asked to blink several times and focus their gaze on the 20/20 line of the chart. They were then instructed to indicate when the line became blurry, namely, when they could no longer read all the letters on the 20/20 line of the Snellen chart. The examiner recorded the time in seconds from the moment the subject opened their eyes to the point at which their visual acuity blurred. This procedure was repeated three times for each eye and the mean value was calculated.

### 2.6. Tear Film Break Up-Time

Tear film break-up time is a valuable method used to assess the stability of the tear film. The procedure was conducted in the following order: a wet fluorescein-impregnated strip was placed in the inferior fornix (I-DEW FLO, Fluorescein Sodium Ophthalmic strips U.S.P., 1 mg (ENTOD Research Cell UK Ltd., London, UK). The patient was then instructed to blink several times to ensure adequate mixing of the dye and to distribute the fluorescein evenly on the ocular surface, and then abstain from further blinking. The TBUT was assessed by measuring the interval between a complete blink and the appearance of the first area of tear film break-up on the cornea, using a broad beam of the slit lamp microscope with a cobalt blue filter. The presence of black spots indicates the existence of dry areas on the tear film. The procedure was repeated three times for each eye and the mean value was recorded. A TBUT of less than 5 s was classified as abnormal [3,10,29]. Novel diagnostic approaches for DED rely on analyzing the precorneal tear film rupture pattern. This method involves observing the appearance and dynamics of dark spots on the front surface of the eye after fluorescein installation. Through careful analysis of this pattern, DED and its subtypes based on predominant etiology can be accurately diagnosed [3,5,30]. 

### 2.7. Statistical Analysis

Statistical analysis was conducted using SPSS 22.0 (IBM Corp., Armonk, NY, USA). Descriptive statistics were used to describe the characteristics of the sample, measures of central tendency, and variability, and were presented as mean values and standard deviations. The frequency distribution for the observed characteristics is presented in both absolute and relative values. All parameters were assessed for normality of distribution. The chi-squared test was used for the comparison of qualitative variables. Levene’s test of equality of variances to test whether samples had equal variances and determine the appropriate t-test. The paired t-test for Equality of Means was used for statistical analysis to examine the differences in TBUT and PBBT of patients with or without symptoms. Correlations between clinical parameters were analyzed by Pearson’s correlation coefficient, aiming to establish the level of interrelationship between the examined variables. *p*-values of <0.05 were considered statistically significant.

## 3. Results

The study involved 62 participants (124 eyes), divided into two groups: Group A, comprising patients with subjective dry eye symptoms, and Group B, including participants without subjective symptoms of DED. Demographic characteristics of the enrolled patients are presented in Table 1. A statistically significant difference based on gender was observed among the examined samples with and without symptoms, *p* < 0.001. 

The majority of patients experiencing symptoms were female (87.10%), while in the asymptomatic group, there was a slight predominance of males (54.84%). In terms of the age distribution among symptomatic patients, the average age was 42 years for women and 48 years for men, whereas for the asymptomatic patients, the average age was 36 years for women and 38 years for men. The predominant age group for both groups encompassed patients between 31 and 50 years of age.

The clinical characteristics of the patients are presented in Table 2. Among patients with symptoms of DED (Group A), no statistically significant difference was observed in the number of subjects with normal and abnormal TBUT values, whereas only eight (12.9%) of asymptomatic patients (Group B) displayed abnormal TBUT values. Furthermore, a significant majority of patients (70.97%) in the symptomatic group (Group A) had a PBBT of 10 s or less. Conversely, in the group of asymptomatic patients (Group B), a higher percentage (61.29%) had PBBT values longer than 10 s. The association between TBUT values and the presence of DED symptoms was investigated, revealing a statistically significant association) (*p* = 0.001). Likewise, the association between PBBT values and the presence of DED symptoms was also found to be statistically significant, *p* = 0.001).

The correlation analysis results between clinical parameters are detailed in Table 3. Pearson’s correlation between TBUT and the total Schein score (Schein questionnaire with additional questions) yielded a statistically significant finding, indicating a moderate negative relationship (r = −0.440, *p* < 0.001). Furthermore, Pearson’s correlation between TBUT and the Schein score (standard Schein questionnaire) consistently revealed a negative relationship, with comparable strength and statistical significance (r= −0.417, *p* < 0.001). Additionally, TBUT values demonstrated a negative correlation with activities requiring focused attention, showing a moderate strength (r = −0.387, *p* < 0.001).

Concerning the age and the results obtained from the Schein questionnaire, a statistically significant and moderately strong positive relationship was observed between age and both the Schein score (r = 0.327, *p* < 0.001) and the total Schein score (r = 0.269, *p* < 0.001). Moreover, the statistical analysis indicated a statistically significant weak and negative correlation between age and TBUT (r = −0.285, *p* < 0.001).

In the analysis of the correlation between PBBT and the evaluated clinical parameters, a statistically significant weak negative association was observed between PBBT and activities requiring focused attention (r = −0.306, *p* < 0.001). Additionally, analogous negative associations were noted with both the total Schein score (r = −0.249, *p* < 0.001) and the Schein score (r = −0.189, *p* < 0.05). Lastly, a statistically significant positive relationship was identified between TBUT and PBBT values (r = 0.455, *p* < 0.001).

The paired t-test for equality of means confirmed a statistically significant difference in the mean TBUT values between patient groups with and without symptoms (*p* < 0.001). Specifically, individuals with symptoms exhibited a mean TBUT value of 4.77 s, while those without symptoms had a mean TBUT value of 7.63 s.

The t-test for equality of means was also used to explore the difference in the mean PBBT values between groups with and without symptoms. The test supported the hypothesis of a statistically significant difference in mean PBBT values for subjects with symptoms (mean = 8.95) compared to those without (mean = 14.66), *p* < 0.001. This suggests that patients experiencing symptoms of DED tend to have shorter PBBT values. Detailed results are presented in Table 4.

**Figure 1:**
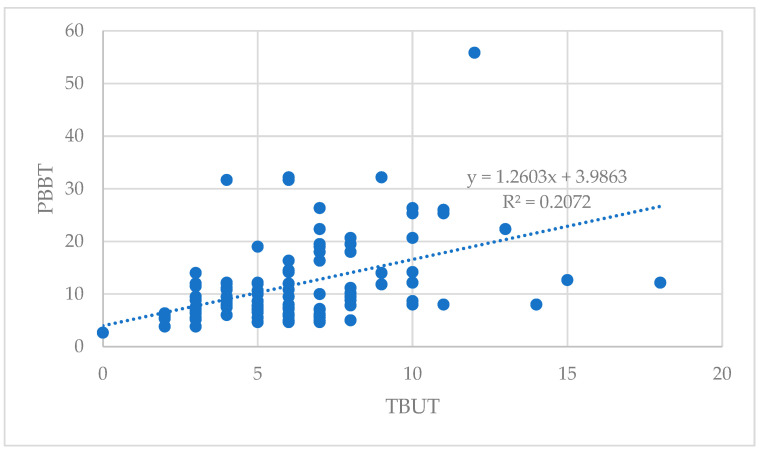
Correlation between PBBT and TBUT.

The correlation between PBBT and TBUT parameters for both eyes is illustrated in the scatterplot diagram (Figure 1). The correlation coefficients for these indicators were 0.46, indicating a statistically significant correlation between PBBT and TBUT.

The PBBT and TBUT showed a statistically significant positive correlation (r  =  0.455; *p* < 0.001; Pearson’s correlation analysis)

## 4. Discussion

Dry eye is a multifactorial disease of the tears and ocular surface with symptoms that often fail to correspond to diagnostic testing [3]. Newer concepts suggest that DED can have a significant impact on visual function influencing daily activities and diminishing overall QoL [7,20,43,44]. Left untreated, the patient may not only experience discomfort and visual disturbances but also face the risk of ocular inflammation, corneal scarring, and permanent damage to the corneal surface [37,44].

The tear film is the foremost refracting surface of the eye that interacts with incoming light and serves as an optical element crucial for maintaining the quality of vision [42,45]. Despite its importance, the comprehensive exploration of the influence of the tear film on optical quality and visual function in dry eyes has been limited. This gap in understanding may be attributed to the common observation that many dry eye patients maintain normal BCVA during routine ophthalmological examinations [15,44,46]. 

In the present study, we explored the impact of tear film dysfunction on visual acuity by measuring TBUT and PBBT in individuals with and without subjective DED symptoms. Our results revealed a significant positive relationship between age and subjective symptoms assessed through the Schein questionnaire. Conversely, we found a significant negative correlation between age and TBUT. The extensive meta-analysis by the International Working Group on Dry Eye (TFOS DEWS II) indicated that dry eye symptoms tend to increase with age, particularly after the age of 50. Furthermore, this study highlighted that the prevalence of dry eye signs increases notably, showing a 10% rise over 10 years, in contrast to a 2% increase in dry eye symptoms during the same period [15] which could be linked to decreased eye surface sensitivity during the aging process. Notably, tear film instability in dry eye syndrome contributes to increased optical aberrations and irregular astigmatism, impacting vision [43]. Our findings also confirmed the correlation between age and subjective symptoms of DED, aligning with prior research [37,38,39,47]. Furthermore, the majority of symptomatic patients were female (87.10%), consistent with the recognized gender-related risk for DED [7,12,15,25].

Visual impairment is common in individuals with dry eyes, especially during tasks requiring focused attention, leading to a reduced blink rate. Despite normal conventional visual acuity, patients with tear film instability may experience fluctuations in visual acuity, impacting activities like driving, reading, and video display terminal use [48,49]. Additionally, it is essential to recognize that heightened levels of eye discomfort and pain also disrupt general activity, mood, sleep, ability to walk, normal work activities, relationships with others, and thereby significantly affecting the patient’s QoL. Therefore, conducting a thorough assessment of both ocular pain and visual function in individuals with DED is needed. This comprehensive evaluation is essential for gaining a deeper understanding of how DED impacts daily life and activities [7]. The Osmoprotection in Dry Eye Disease–Expert Opinion (OCEAN) group highlighted challenges in daily tasks for patients with DED [50]. Complaints of blurred vision, glare, and fluctuating vision are common, even with normal BCVA. A correlation has been noted between the severity of DED and HOA levels, where tear film instability contributes to post-blink HOA, resulting in vision fluctuations and increased blinking frequency. The presence of irregular astigmatism and HOA is associated with a decline in contrast sensitivity, a key element of normal visual function [43,51]. Despite this connection, the absence of established evaluation methods presents a challenge in objectively linking optically assessed quality to subjective visual symptoms in dry eyes. It is essential to identify and employ reliable techniques for accurately measuring visual functions related to dry eye symptoms [52,53,54,55].

Our study combined the modified Schein questionnaire with TBUT and PBBT tests, revealing statistically significant data regarding impaired visual function in DED patients. These findings suggest that TBUT and PBBT can offer valuable clinically relevant data, complementing established dry eye questionnaires. We found a statistically significant negative correlation between PBBT values and subjectively assessed dry eye symptoms, as well as activities requiring focused attention. Conversely, a significant positive correlation was observed between TBUT and PBBT values.

There are several advantages of PBBT over TBUT in clinical practice. TBUT is an invasive technique involving a wet fluorescein-impregnated strip or a drop of a 1% or 2% fluorescein solution. The paper strip’s contact with the eye induces reflex lacrimation, whereas instilling a fluorescein solution, even just one drop, exceeds the tear volume by three to six times, resulting in excessive destabilization of the tear film. Moreover, adding fluorescein to the tear film alters the physical interactions between the layers, reducing surface tension and consequently affecting the TBUT value [56]. Additionally, the fluorescein dye stains soft lenses, necessitating their removal before staining, which can be an inconvenience for the patient. In contrast, PBBT allows the non-invasive, simpler, and more accurate assessment of tear film stability [3].

Spontaneous blinking, crucial for distributing the tear film on the ocular surface is considered to be essential for optimal optical quality. Individuals with DED cope with blurred vision by increasing blink rates to redistribute the tear film [43]. If blinking is not rapid enough, retinal image quality is compromised [7]. Goto et al. [57] validated this, observing a decline in visual acuity of 0.3 in 16 patients with dry eyes after keeping their eyes open for only 10 s. It is important to highlight that even in normal subjects, tear film optical quality can deteriorate with an interblink interval longer than 10 s [58].

Various methods have been employed to assess DED, such as measuring TBUT, cornea fluorescence score, Rose Bengal staining, Schirmer test, and tear osmolarity measurement [10,28,59]. Recent research has demonstrated that alterations in visual function associated with DED are manifestations of tear film instability, highlighting the interconnection of visual and tear film quality assessment. Talens-Esteralles et al.’s study provided valuable insights into visual quality both objectively and quantitatively through the analysis of light disturbance, revealing that various aspects of visual function and quality deteriorated among computer users through the course of the day. Additionally, D’Souza et al. found that patients with DED and unstable tear film exhibited significantly poorer quality of vision and optical optics, exerting a notable influence on their QoL. However, their comprehensive research relied on the use of specialized and sophisticated equipment not commonly available in routine ophthalmological practice [20,21]. However, many of these assessments require specific reagents and equipment. A significant challenge in ophthalmology practice has been the lack of objective, non-invasive, and reproducible screening tests for DED. The development of such tests could prove instrumental in early diagnosis and contribute to enhanced long-term outcomes. Several studies have attempted to assess the impact of dry eye on visual function in a straightforward manner, suitable for conducting under everyday conditions and without relying on specialized or sophisticated equipment [10,58,60,61,62]. A recently conducted study assessed the discriminatory capability of the blink test in identifying individuals with DED. The blink test measures the time it takes, following two non-forceful blinks, for a patient to experience symptoms of ocular discomfort or dry eye. Utilizing a cut-off time of 10 s, the blink test demonstrated a sensitivity of 66%, and specificity of 88%, in predicting a diagnosis of DED according to the TFOS DEWS II criteria. In the same investigation, the correlation between the blink test and non-invasive TBUT was r = 0.47 (*p* < 0.001). When integrated with the screening questionnaire, the sensitivity and specificity of the blink test increased to 71% and 90%, respectively. Similarly, Hwang et al. [60] introduced the blinking tolerance time (BTT) test, which assesses tear film stability indirectly by measuring the time interval between eye blinks when the participant refrains from blinking for as long as possible until experiencing a foreign body sensation or irritation. They discovered a notable correlation between BTT and TBUT, with BTT values being significantly shorter in dry eye patients compared to healthy subjects. Inomata et al. [10] investigated the usefulness of the maximum blink interval (MBI) and the blink interval period (BIP) in screening for DED. MBI represents the time in seconds that participants can keep their eyes open and BIP denotes the duration between the appearance of dark spots on the ocular surface and the subsequent blink. As such it represents the difference of values between MBI and TBUT in seconds. BIP was found to be diminished in the DED group compared to the non-DED group of patients, suggesting that tear film layer instability in DED not only reduces TBUT but also diminishes the period between dark spot occurrence and blinking.

In our investigation, we used the PBBT test for evaluating tear film instability by measuring the time interval between eye blinks when the participant has been asked to refrain from blinking until the point when vision becomes blurred. Our study demonstrated a significantly strong correlation between PBBT and TBUT (r = 0.455, *p* < 0.001), highlighting the influence of tear break-up on corneal light refraction. This effect, in turn, compromises the quality of vision and contributes to a reduction in visual acuity. The PBBT test is a cost-effective diagnostic tool that clinicians can readily use as a widespread screening method for detecting DED without the need for the use of sophisticated equipment. Additionally, it may serve as a simple method for self-diagnosis. Our results indicate that the PBBT test may serve as a valuable supplementary diagnostic tool for dry eye, complementing the TBUT test. Additionally, we analyzed the correlation between the PBBT and subjectively assessed dry eye symptoms, which yielded a significantly negative correlation. These results verify the association of the PBBT test with objective signs as well as subjective symptoms of dry eye. The PBBT test may have many advantages. Specifically, it can be used as a self-test for dry eye disease which is very easy and simple to perform without the need for assistance from a medical professional. It provides insight into the physiological condition of the tear film without requiring fluorescein dye for the testing process. Additionally, the test results remain unaffected by an individual’s pain threshold, a factor that could influence tests relying on ocular pain as the endpoint. This could be particularly important when testing the aging population due to the additional decline in sensitivity of the eye surface during the aging process.

This research describes the impact of tear film dysfunction on visual acuity and introduces PBBT as a potential non-invasive tool for assessing tear film stability. A new concept for evaluating tear film stability by determining visual function is proposed. The main finding of this study was that PBBT, dry eye symptoms, and TBUT were related. PBBT showed a significant positive correlation with TBUT and a significant negative correlation with subjective dry eye symptoms, highlighting its potential as a promising and clinically relevant diagnostic tool to identify individuals with DED. This confirms the idea that the stability of the tear film plays an important role in refracting light and maintaining the optical quality of vision. Using PBBT, tear film stability can be assessed in a non-invasive, straightforward uncomplicated way. The PBBT test is simple to perform, applicable in everyday ophthalmological practice, requires no additional reagents or equipment, and is time-efficient without necessitating additional financial resources or training for implementation. A significant advantage is the fact that the test is non-invasive, eliminating the need for eye drops or physical contact with the eye, which could potentially influence results. Additionally, it is suitable for use during optometric examinations, can serve as a self-examination method, and is beneficial for patients with a fear of eye drops. The PBBT test holds promise as a widespread screening method for DED, which could be further confirmed with more extensive ophthalmological examinations utilizing specialized equipment and reagents.

Finally, it is important to note that this study has several limitations. Patients included in the study were classified based solely on subjective symptoms and responses to the Schein questionnaire. While clinical examination and objective signs of DED were not criteria for inclusion, they were, however, part of the overall clinical evaluation in the study. Clinical data showed that only 12.9% of patients who did not exhibit any subjective symptom of dry eye had pathological TBUT values of less than 5 s, and the mean TBUT value in this group was 7.63 ± 3.25, significantly higher than that in the group of patients experiencing subjective symptoms of dry eye. Another potential limitation may be the use of the Schein questionnaire rather than opting for a more comprehensive tool, such as the ocular surface disease index (OSDI) questionnaire. The Schein questionnaire lacks a validated cut-off value for dry eye, representing a significant limitation. This absence of a standardized cut-off value could potentially impact the classification of patients into the studied groups. Moreover, the Schein questionnaire does not evaluate the impact of DED on factors such as quality of vision, QoL, or daily activities, as comprehensively captured by the OSDI questionnaire. In response to this limitation, we included additional questions in the survey. On a positive note, Schein’s questionnaire was selected for its frequent and straightforward application in daily clinical practice. It proves to be practical, concise, and easily understandable for all patients, particularly the elderly and is not protected by copyright [16,33,63]. Moreover, it is crucial to recognize that correcting refractive errors, particularly in individuals with cataracts and high astigmatism, can influence visual function and, subsequently, the outcomes of the PBBT test, thereby introducing potential additional limitations [31,41,46,56]. Nevertheless, the study recruited only participants with a refractive error of up to 1 spherical diopter, excluding individuals with astigmatism or cataracts. Consequently, we believe that any potential impact on test results due to refractive error and subsequent aberration was minimal. 

## 5. Conclusions

Dry eye, a prevalent and complex ocular surface disease, causes discomfort, blurred vision, reduced quality of life, and decreased productivity. Our study emphasizes the significant role of tear film stability in visual function. The PBBT test holds promise as an objective and clinically significant screening tool for individuals with undiagnosed DED. The escalating global prevalence of DED emphasizes the importance of early prevention and self-management to uphold the quality of vision and overall quality of life.

## Figures and Tables

**Table 1:** Demographic characteristics of the patients.

		Symptoms			
		Group A Symptomatic Patients *n* (%)	Group B Asymptomatic Patients *n* (%)	Total *n* (%)	*p*-Value
Gender	Male	8 (12.90)	34 (54.84)	42 (33.87)	<0.05
	Female	54 (87.10)	28 (45.16)	82 (66.13)	
	Total	62 (100)	62 (100)	124 (100)	
Age (years)	15–30	20 (32.26)	22 (35.48)	42 (33.87)	NS
	31–50	24 (38.71)	28 (45.16)	52 (41.94)	
	≥51	18 (29.03)	12 (19.35)	30 (24.19)	
	Total	62 (100)	62 (100)	124 (100)	

*n*—number, NS—non-significant.

**Table 2:** Clinical characteristics of the patients.

		Symptoms			
		Group A Symptomatic Patients *n* (%)	Group B Asymptomatic Patients *n* (%)	Total *n* (%)	*p*-Value
TBUT	<5	32 (51.61)	8 (12.90)	40 (32.26)	<0.001
	≥5	30 (48.39)	54 (87.10)	84 (67.74)	
	Total	62 (100)	62 (100)	124 (100)	
PBBT	≤10	44 (70.97)	24 (38.71)	68 (54.84)	<0.001
	>10	18 (29.03)	38 (61.29)	56 (45.16)	
	Total	62 (100)	62 (100)	124 (100)	

TBUT—tear film break-up time, PBBT—post-blink blur time, *n*—number.

**Table 3:** Correlations between the clinical parameters analyzed by Pearson’s correlation coefficient.

	Age	Schein Score (Schein Questionnaire)	TBUT	Activities Requiring Focused Attention	Total Schein Score (Schein Questionnaire + Additional Questions)	PBBT
Age	1	0.327 **	−0.285 **	0.022	0.269 **	0.038
Schein score (Schein questionnaire)	0.327 **	1	−0.417 **	0.695 **	0.967 **	−0.189 *
TBUT	−0.285 **	−0.417 **	1	−0.387 **	−0.440 **	0.455 **
Activities requiring focused attention	0.022	0.695 **	−0.387 **	1	0.830 **	−0.306 **
Total Schein score (Schein questionnaire + additional questions)	0.269 **	0.967 **	−0.440 **	0.830 **	1	−0.249 **
PBBT	0.038	−0.189 *	0.455 **	−0.306 **	−0.249 **	1

** Correlation is significant at the 0.01 level (2-tailed). * Correlation is significant at the 0.05 level (2-tailed). TBUT—tear film break-up time, PBBT—post-blink blur time.

**Table 4:** Statistical differences in TBUT and PBBT values in patients with and without symptoms of DED.

		*n*	Mean	Standard Deviation	Standard Error Mean	*p*-Value
TBUT	Group A Symptomatic patients	62	4.77	2.37	0.30	<0.001
	Group B Asymptomatic patients	62	7.63	3.25	0.41	
PBBT	Group A Symptomatic patients	62	8.95	5.38	0.68	<0.001
	Group B Asymptomatic patients	62	14.66	10.50	1.33	

TBUT—tear film break-up time, PBBT—post-blink blur time, *n*—number.

## Data Availability

Data is contained within the article.
